# *Cryptosporidium parvum* rhomboid1 has an activity in microneme protein CpGP900 cleavage

**DOI:** 10.1186/s13071-016-1728-6

**Published:** 2016-08-08

**Authors:** Mingying Li, Xichen Zhang, Pengtao Gong, Jianhua Li

**Affiliations:** Key Laboratory of Zoonosis, Ministry of Education, College of Veterinary Medicine, Jilin University, Changchun, 130062 China

**Keywords:** *Cryptosporidium parvum*, Rhomboid, Microneme, Interaction

## Abstract

**Background:**

Apicomplexan parasites actively release transmembrane (TM) adhesive proteins involved in host cell attachment and invasion. Rhomboids, a family of intramembrane serine proteases, cleave these secreted adhesive proteins within their TM domains as an essential step in completing the invasion process. In *Cryptosporidium parvum*, the activity of rhomboids in cleaving microneme proteins (MICs) has not been reported. In the present study, the interaction between *C. parvum* rhomboids (CpROM1 and CpROM4) and *C. parvum* microneme proteins (CpGP900 and CpTRAP-C1) was investigated using yeast two-hybrid assay and co-immunoprecipitation assays.

**Results:**

Our study demonstrated that CpROM1 protein could interact with CpGP900 protein in co-transformed AH109 yeasts. Analysis of these proteins in co-transfected mammalian cells showed that the cleavage product of the CpGP900 protein was detected in the co-transfected cells. As control, CpGP900 only was transfected into cells and no cleavage was observed. The results suggested that CpGP900 protein was the substrate of CpROM1. Moreover, CpROM1 and CpROM4 could not cleave CpTRAP-C1 protein, which is the substrate of *T. gondii* rhomboid 2.

**Conclusions:**

Our results showed that CpROM1 is an active protease that is involved in microneme protein CpGP900 cleavage, which lay the foundation for further research on the mechanisms of *C. parvum* invasion.

## Background

*Cryptosporidium parvum* is an obligate intracellular pathogen belonging to the phylum Apicomplexa which infects the microvillus border of the gastrointestinal epithelium of a wide range of animals, including humans [1]. Most apicomplexan parasites invade host cells by an active process dependent on actinomyosin-powered gliding motility [2, 3]. The invasive stages involve specialized apical organelles, called micronemes and rhoptries, whose sequential secretions are required for successful penetration of the host plasma membrane [4]. Micronemes, the smallest apical secretory organelles, release several adhesive proteins from the apical end of the parasite onto the surface to establish specific interactions with host cell receptors and power parasite gliding motion by connecting to the actomyosin system of the parasite during the process of invasion [5, 6]. Upon release onto the host cell surface, microneme proteins (MICs) with transmembrane domains (TMDs) are transported to the posterior end of the parasite and are eventually cleaved off the surface of the parasite to complete the penetration process [7]. The rhomboids are a well-conserved family of intramembrane serine proteases which are implicated in growth factor signaling, mitochondrial function, and host cell invasion by apicomplexan parasites [8]. Research indicates that rhomboid proteins have the unique characteristic of cleaving cell surface adhesion proteins and play a part in apicomplexan parasite invasion [9]. In *Toxoplasma gondii* and *Plasmodium falciparum*, processing of key microneme adhesins, such as TgMIC2, TgMIC6, TgAMA1 and PfEBA175, has been shown to occur via intramembranous cleavage at sites predicted to be rhomboid-like substrates [10–13]. The intramembrane cleavage site in TgMIC2 and TgMIC6 has been mapped by mass spectrometry to the site IA↓GG [11], which is conserved in other apicomplexan transmembrane MICs.

In *C. parvum*, the substrate and its role in microneme proteins cleavage of rhomboid-like proteins were unknown. *Cryptosporidium parvum* ROM1 (GenBank accession: BAJ77699), ROM4-1 (GenBank accession: EAK90309) and ROM4-2 (GenBank accession: EAK89768) were first identified using basic local alignment search tool (BLAST) and named according to their clustering in phylogenetic analysis [14]. Although the cleavage characteristics of ROMs have been described in other apicomplexan parasites, the function of CpROMs is not clear.

MICs of *C. parvum* have been identified including CpGP900, CpTRAP-C1 and CpMIC1. According to sequence analysis, CpGP900 and CpTRAP-C1 have the intramembranous cleavage site and were predicted to be rhomboid substrates. CpGP900 is a mucin-like glycoprotein with a putative transmembrane domain, which is stored in micronemes prior to appearance on the surface of *C. parvum* during the invasive stages and is shed from their surface [15]. Previous studies also showed that purified native CpGP900 competitively inhibited *C. parvum* infection of MDCK cells in vitro, suggesting that CpGP900 mediates attachment and invasion [15]. The thrombospondin (TSP)-related adhesive protein of *Cryptosporidium*-1 (CpTRAP-C1), a member of the TSP-related protein family of apicomplexan parasites, is localized in the apical end of *C. parvum* sporozoites and is structurally related to the micronemal proteins TgMIC2, which is involved in host-cell attachment and invasion [16].

In the present study, the potential of CpMICs (CpGP900 and CpTRAP-C1) to interact with and to be cleaved by *C. parvum* rhomboids (CpROM1 and CpROM4) was examined by yeast two-hybrid and co-immunoprecipitation assays.

## Methods

### Parasites, yeast strain and cell culture

*C. parvum* oocysts were originally obtained from naturally infected calves from Changchun in China and were stored at 4 °C in 2.5 % potassium dichromate (K_2_Cr_2_O_7_) aqueous solution until use. Yeast strain AH109 was cultured at 30 °C in YPDA medium. Hela cells were cultured in RPMI 1640 supplemented with 10 % FBS at 37 °C in a 5 % CO_2_ incubator. The cells were seeded in six-well plates at a density of 3 × 10^5^ per well.

### Bait plasmid construction and auto-activation detection

Yeast two-hybrid screening was performed with the Matchmaker™ GAL4 two-hybrid system (Clontech, Palo Alto, CA, USA). The open reading frame (ORF) encoding *C. parvum* rhomboid 1 (CpROM1: GenBank FX115596.1) and *C. parvum* rhomboid 4 (CpROM4: Gene ID 3372282) were amplified by PCR with forward primer (CpROM1: 5′-ATG TCA AAT ATA CAC AG-3′; CpROM4: 5′-ATG TCT GAC AGA AAG ATT-3′) and reverse primer (CpROM1: 5′-TCA AGG ATT CAT AAG T-3′; CpROM4: 5′-TTA TCC ACA TCT TCT AAT CC-3′). The DNA fragments were inserted into the pGBKT7 vector digested with NcoI/SalI as the baits. The ORF encoding *T. gondii* rhomboid 2 (TgROM2: GenBank AY704176.1) was amplified by PCR (forward primer 5′-ATG GCC AAC ATT CGG AC-3′; reverse primer 5′-TCA GCA GCG AGG GAC CA-3′) from *T. gondii* cDNA and inserted into pGBKT7 digested with EcoRI/SalI.

Using LiAc-mediated yeast transformation, the bait plasmid pGBKT7-CpROM1, pGBKT7-CpROM4 or pGBKT7-TgROM2 was separately transformed into yeast strain AH109 and spread on the selection plate lacking histidine. Plasmids pCL1 and pGBKT7 were also transformed into AH109 as the positive control and negative control, respectively. The autonomous activity of the bait plasmids were detected by β-galactosidase activity assay (added X-α-gal to the plates at 20 μg/ml).

### Yeast two-hybrid screening

The DNA fragments encoding A-T domain (amino acids Ala1641-Thr1816) of CpGP900 (GenBank: AF068065.1) and I-S domain (amino acids Ile148-Ser687) of CpTRAP-C1 (GenBank: AF017267.1) which contains the TM domains were amplified by PCR (CpGP900: forward primer 5′-GGA ATT CGC TGG ATT CAT TTC TGG T-3′, reverse primer 5′-CGG GAT CCC GTT CCA GAA TGA TGA A-3′; CpTRAP-C1: forward primer 5′-GGA ATT CAT CGG AGA GTG TGC GAC AAC AT-3′, reverse primer 5′-CCC TCG AGT CAA CTA GCC CAG TTC TGA CTC-3′) and inserted into the EcoRI/XhoI site or EcoRI/BamHI site of pGADT7 as the preys. According to the manufacturer’s instructions, the bait plasmids and prey plasmids, in the following combinations, were co-transformed into the yeast AH109 strains: pGBKT7-CpROM1 and pGADT7-CpGP900; pGBKT7-CpROM4 and pGADT7-CpGP900; pGBKT7-CpROM1 and pGADT7- CpTRAP-C1; pGBKT7-CpROM4 and pGADT7-CpTRAP-C1; pGBKT7-TgROM2 and pGADT7-CpTRAP-C1; pGBKT7-53 and pGADT7-T (positive control); pGBKT7-Lam and pGADT7-T (negative control). All co-transformed yeasts were cultured on the SD/-Trp/-Leu plates at 30 °C. Interactions between bait and prey proteins were confirmed via colony growth in selection medium SD/-Trp/-His/-Leu/-Ade. Positive clones were verified using the β-galactosidase activity assay.

### Verification of the interaction by co-immunoprecipitation

The cDNA sequences of CpROM1, TgROM2 were subcloned into pcDNA3.1-myc vector and CpGP900, CpTRAP-C1 were subcloned into pcDNA3.1-HA vector. Hela cells were cultured in DMEM plus 10 % FBS in a 6-well plate at a density of 3 × 10^5^/well one day before transfection. The cells were transfected with plasmids pcDNA3.1-myc-CpROM1, pcDNA3.1-myc-TgROM2, pcDNA3.1-HA-CpGP900, pcDNA3.1-HA-CpTRAP-C1, pcDNA3.1-myc-CpROM1 and pcDNA3.1-HA-CpGP900, pcDNA3.1-myc-TgROM2 and pcDNA3.1-HA-CpTRAP-C1, respectively, using Lipofectamine 2000 (Invitrogen, Carlsbad, CA, USA) according to the manufacturer’s instructions.

After 48 h, the transfected cells were collected and lysed on ice in RIPA lysis buffer (Sangon Biotech, Shanghai, China) for 20 min. The lysate was incubated overnight at 4 °C with anti-HA or anti-myc antibody, and then precipitated by Protein A agarose (Invitrogen, Carlsbad, CA, USA). Bound proteins were detected by Western blotting using anti-HA or anti-myc antibody (Proteintech, Chicago, USA).

## Results

### CpROM1 interacted with CpGP900 protein and TgROM2 interacted with CpTRAP-C1 protein in yeast two-hybrid assay

AH109 yeasts transformed with bait plasmids pGBKT7-CpROM1, pGBKT7-CpROM4, pGBKT7-TgROM2 did not turn blue after β-galactosidase assay, indicating that CpROM1, CpROM4 and TgROM2 did not activate GAL4 reporter gene (Table 1).

**Table 1:** Evaluation of the autoactivation of bait plasmids

Bait plasmid	Growth on SD/-Leu or SD/-Trp plate	β-galactosidase activity
pCL1	+	+
pGBKT7-CpROM1	+	–
pGBKT7-CpROM4	+	–
pGBKT7-TgROM2	+	–
pGBKT7	+	–

+, growth and/or β-galactosidase activity; –, no growth and/or no β-galactosidase activity

In co-transformation groups, yeast cells transformed with pGBKT7-CpROM1 and pGADT7-CpGP900 could grow on SD/-Trp/-His/-Leu/-Ade plates, and the colonies turned blue in β-galactosidase activity assay. Yeasts co-transformed with pGBKT7-CpROM4 and pGADT7-CpGP900 did not grow on SD/-Trp/-His/-Leu/-Ade selection plates (Table 2). Yeasts co-transformed with pGBKT7-TgROM2 and pGADT7-CpTRAP-C1 grew on SD/-Trp/-His/-Leu/-Ade plates and colonies were also positive in β-galactosidase assay. The yeast cells co-transformed with pGBKT7-CpROM1 and pGADT7-CpTRAP-C1 or pGBKT7-CpROM4 and pGADT7-CpTRAP-C1 did not grow on SD/-Trp/-His/-Leu/-Ade plates (Table 2).

**Table 2:** Interactions of CpROMs, TgROM2 and MIC proteins by yeast two-hybrid assay

Bait plasmid	Prey plasmid	Growth on selection plate	β-galactosidase activity
pGBKT7-53	pGADT7-T	+	+
pGBKT7-CpROM1	pGADT7-CpGP900	+	+
pGBKT7-CpROM4	pGADT7-CpGP900	–	–
pGBKT7-CpROM1	pGADT7-CpTRAP-C1	–	–
pGBKT7-CpROM4	pGADT7-CpTRAP-C1	–	–
pGBKT7-TgROM2	pGADT7-CpTRAP-C1	+	+
pGBKT7-Lam	pGADT7-T	–	–

+, growth and/or β-galactosidase activity; –, no growth and/or no β-galactosidase activity

### CpROM1 and TgROM2 could cleave CpGP900 and CpTRAP-C1, respectively, in mammalian cells

In the co-transfection group of pcDNA3.1-myc-CpROM1 and pcDNA3.1-HA-CpGP900, a smaller CpGP900 protein band of approximately 14 kDa was detected with anti-HA antibody in anti-myc immunoprecipitates. As control, a band of size 19 kDa corresponding to CpGP900 was observed in the transfection group of pcDNA3.1-HA-GP900 only and no cleavage product was detected. Furthermore, the CpROM1 protein band of size 32 kDa was detected when co-transfected cells lysates were immunoprecipitated with anti-HA antibody, but not detected in control precipitates. In the co-transfection group of pcDNA3.1-myc-TgROM2 and pcDNA3.1-HA-CpTRAP-C1, the cleavage product of the CpTRAP-C1 protein of approximately 52 kDa was detected with anti-HA antibody when cells lysates were immunoprecipitated with anti-myc antibody. However, pcDNA3.1-HA-CpTRAP-C1 only was transfected into cells and no cleavage protein was observed. Meanwhile, the TgROM2 band of size 31 kDa appeared in anti-HA immunoprecipitates by Western blotting with anti-myc antibody. These results suggest that CpGP900 could be cleaved by CpROM1 and CpTRAP-C1 could be the substrate of TgROM2, as evidenced by the appearance of the cleaved CpGP900 and CpTRAP-C1 products in co-transfection groups.

## Discussion

The rhomboids are a family of intramembrane serine proteases that are well-conserved across evolution. *Drosophila melanogaster* Rhomboid-1 was the first member of this protease family described [17] and was responsible for cleaving Spitz, which leads to the secretion of the active epidermal growth factor receptor ligand [18]. Since then, the rhomboid-like genes have been discovered throughout all kingdoms of life. These proteins are implicated in diverse biological processes, such as quorum sensing in bacteria, mitochondrial membrane fusion, apoptosis, stem cell differentiation in eukaryotes, human diseases and parasitic diseases [19].

In apicomplexans, rhomboid-like genes have been reported in many parasites, including *T. gondii*, *Plasmodium* spp., *Eimeria tenella*, *Cryptosporidium* spp., *Neospora caninum*, *Babesia bovis* and *Theileria* spp. Dowse & Soldati [14] first performed BLAST searches using *D. melanogaster* Rhomboid-1 and human RHBDL2 to identify several rhomboid-like genes in parasite sequences. In addition, the rhomboids were named based on their clustering in phylogenetic analysis as the rhomboids within a cluster may share similar functions. According to the studies of several rhomboids of *T. gondii* and *Plasmodium* spp., the different rhomboid proteases have distinct subcellular locations and expressed at different developmental stages with different functions [20].

Recent research revealed that an important role of the rhomboid-like proteases is participating in shedding adhesive proteins from the surface of the parasites during motility and invasion. Initial insights into rhomboid function in apicomplexan parasites came from studies on the micronemal protein protease 1 (MPP1). In *T. gondii*, MPP1 cleaved TgMIC2, TgMIC6, TgMIC12 and TgAMA1 within their TMDs, which are implicated in distinct steps of the invasion process [11, 21–23]. Studies also suggested that rhomboid-like proteins are implicated as candidates for MPP1 while the rhomboids could cleave the TMDs resulting in the release of their substrates [24, 25]. TgROM1, a micronemal protein, is implicated in intracellular parasite growth [26] and is active in vitro against Spitz [27]. Meanwhile PfROM1 could cleave PfAMA1, RBL proteins and EBL adhesion [28]. ROM2, located to the Golgi apparatus, has been found only in *N. caninum* and *T. gondii*. TgROM2 could cleave chimeric proteins that contain the TMDs of TgMIC2 and TgMIC12 [29]. Recent studies verified that *E. tenella* rhomboid 3 was an active protease in the cleavage of EtMIC4 [30]. The biological function and substrate of ROM3 in other parasites have not been reported. TgROM4 and PfROM4 localize to the plasma membrane of extracellular parasites [13, 27]. TgROM4 was considered inactive in the cell-based cleavage assay [27], but conditional knockout of TgROM4 demonstrated that this protease is essential in processing of surface adhesins including MIC2, AMA1 and MIC3 [31]. PfROM4 could cleave a wide range of *Plasmodium* adhesins, such as EBA-175, RBL proteins and each EBL adhesion [28]. TgROM5 was supposed to be highly active against the full-length MIC2 adhesin and the TMDs of MIC6 and MIC12 [27].

**Fig. 1:**
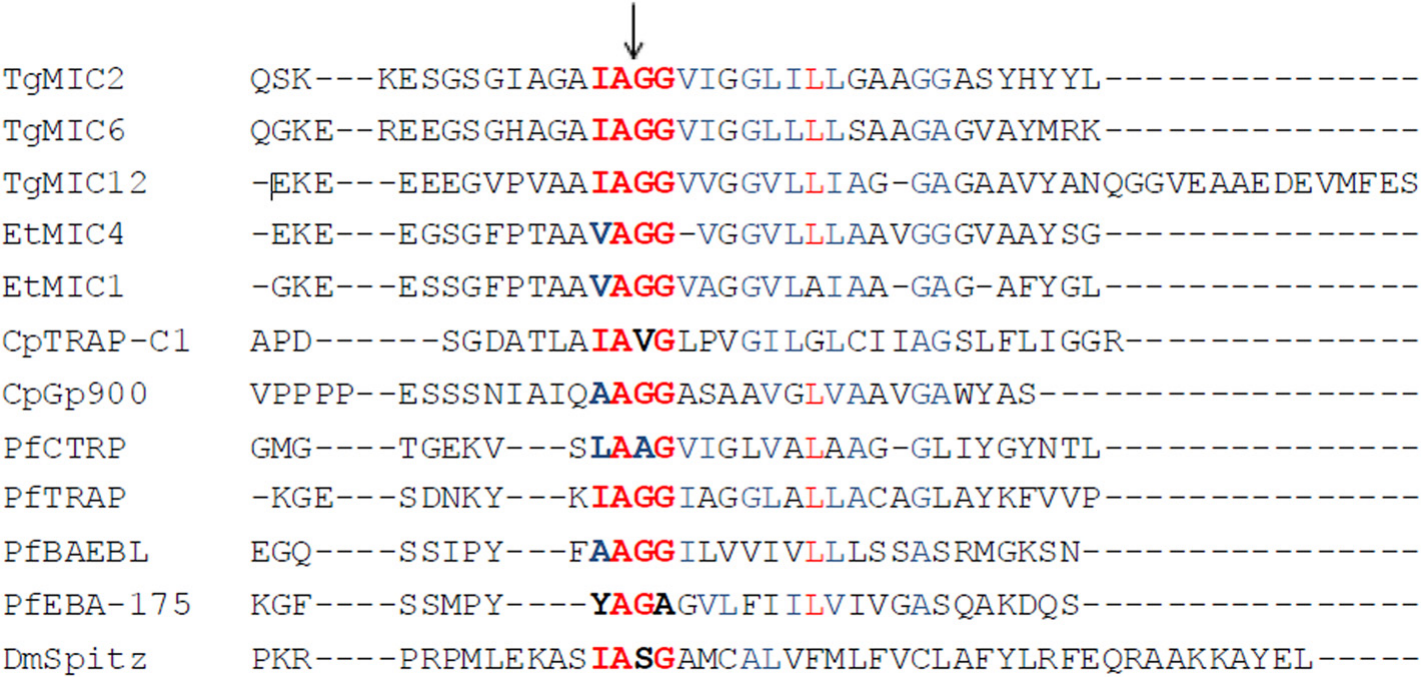
Amino acid sequence alignment of the TM domains of the apicomplexan microneme proteins. TgMIC2 (GenBank AAB63303), TgMIC6 (GenBank AAD28185), TgMIC12 (GenBank AAK58479), EtMIC4 (GenBank CAC34726), EtMIC1 (GenBank 043981), CpTRAP-C1 (GenBank EAK88310), CpGP900 (GenBank AAC98153), PfCTRP (GenBank NP-473263), PfTRAP (GenBank AAB63302), PfBAEBL (GenBank NP-705031), PfEBA-175 (GenBank AAP76315), DmSpitz (GenBank Q01083). Conserved and identical residues are shown in *red text*. Similar residues are shown in *blue text*. The cleavage sites of TgMIC2 and TgMIC6 are shown by a *black arrow. Abbreviations*: Tg, *Toxoplasma gondii*; Et, *Eimeria tenella*; Cp, *Cryptosporidium parvum*; Pf, *Plasmodium falciparum*; Dm, *Drosophila melanogaster*

In *C. parvum*, only rhomboid 1 and 4 have been identified and sequenced until now, while rhomboid 4 contains two members: ROM4-1 (EAK90309) and ROM4-2 (EAK89768). But the activity and substrate of *C. parvum* rhomboids had not been reported. Here the interaction of CpROMs and CpMICs was studied by yeast two-hybrid and co-immunoprecipitation assays. As shown in Fig. 1, the C-terminal membrane-spanning domains and cytoplasmic domains of *T. gondii*, *P. falciparum*, *E. tenella* and *C. parvum* MICs share a striking conservation of the amino acid sequences which harbor the conserved cleavage site. In *C. parvum*, both CpTRAP-C1 and CpGP900 proteins, the two members of the MICs that participate in the process of host cell invasion, have the conserved TMDs and were likely to be rhomboid substrates.

To reveal the substrates of CpROMs, a yeast two-hybrid system was used to detect the interaction of the *C. parvum* ROMs and MICs. In this study, the interaction between CpROM4-1 and MICs was not explored on account of the failure of the expression of CpROM4-1 (EAK89768) in the eukaryotic cells. Thus, the cleavage activity of only *C. parvum* ROM1 and ROM4-2 were detected in the experiment. The results indicated that only CpGP900 could be cleaved by CpROM1 in vitro using the yeast two-hybrid assay. Meanwhile, the interaction of the two proteins was further validated by co-immunoprecipitation and western blotting. The cleaved CpGP900 protein band suggested that the CpGP900 protein was the substrate of *C. parvum* ROM1. According to our results, despite CpROM4 being is inactive in cleaving CpGP900 and CpTRAP-C1 in vitro, it could participate in other processes or have other substrates that have yet to be discovered. It has been shown that distantly related rhomboid proteases can cleave the same synthetic model substrates at identical positions [32], and rhomboids of one species can cleave substrates from another species. TgROM1, TgROM2, TgROM5 and PfROM1 cleave the TM domain of *Drosophila* Spitz, an established substrate for rhomboids of several species [28, 29]. TgROM5 could cleave both PfAMA1 and PfBAEBL efficiently within their TMDs [28]. And in our study, CpTRAP-C1 could be cleaved by TgROM2, but not by CpROMs 1or 4. These data suggest that some rhomboids display broad substrate specificities and cleave diverse substrates.

In apicomplexans, the cleavage of MICs within their TMDs is a critical step to invade the host cells effectively. Our study revealed that CpROM1 is active in cleaving CpGP900 protein and may play a crucial role during host invasion; this justifies further efforts to identify this protease as a potential drug target against *C. parvum* infection. Meanwhile, the results lay the foundation for further research on the mechanisms of *C. parvum* invasion. However, the physiological function of CpROMs remains unknown and a genetic approach is required to confirm whether the rhomboids are essential for invasion. The questions may be answered by conditional disruption of the ROM genes.

## Conclusions

In conclusion, the present study suggests that *C. parvum* ROM1 interacts with *C. parvum* MIC protein GP900 and is also involved in the cleavage of CpGP900, which play a crucial role during host invasion. These results may be helpful in further research on the mechanisms of *C. parvum* invasion. However, further research on the detailed process of *C. parvum* rhomboids in cleaving MICs is still needed.

## Abbreviations

BLAST, basic local alignment search tool; MICs, microneme proteins; MPP1, micronemal protein protease 1; ORF, open reading frame; ROM, rhomboid; TM, transmembrane; TMD, transmembrane domain; TRAP-C1, thrombospondin-related adhesive protein of *Cryptosporidium*-1; TSP, thrombospondin.
